# Nanobodies as antivirals against rabies in experimentally infected mice

**DOI:** 10.1590/S1678-9946202567063

**Published:** 2025-10-03

**Authors:** Washington Carlos Agostinho, Viviana Parreño, Celina Guadalupe Vega, Matias Aduriz, Carolina Moura de Oliveira, Sheila Olivera de Sousa Silva, Joana Aguiar, Sueli Akemi Taniwaki, Paulo Eduardo Brandão

**Affiliations:** 1 Universidade de São Paulo, Faculdade de Medicina Veterinária e Zootecnia, Departamento de Medicina Veterinária e Preventiva e Saúde Animal, São Paulo, São Paulo, Brazil; 2 Instituto Nacional de Tecnología Agropecuaria, Buenos Aires, Argentina

**Keywords:** Rabies, Nanobodies, Antiviral treatment

## Abstract

Despite its 100% lethality and approximately 59,000 human deaths every year, rabies still lacks an effective treatment. Numerous trials have aimed to impair the life cycle of *Lyssavirus rabies* (RABV), the primary worldwide lyssavirus causing rabies, but with limited success. Treatments targeting host factors and attempting to mitigate the damage caused by RABV have also been unsatisfactory. This article describes the effects of intracerebral transfection of anti-RABV recombinant monoclonal nanobodies as antivirals against rabies *in vivo*, in a post-exposure protocol. Mice were intranasally inoculated with the RABV CVS strain and, 72 h later, were injected via the intracerebral route with two different anti-RABV llama-derived VHH nanobodies complexed with a transfection agent. One of the VHHs was able to reduce the viral load in mice, but no significant effect on survival was detected. Though not completely effective, nanobody therapy could be attempted in association with other antivirals to improve therapies against rabies.

## INTRODUCTION

Rabies is a zoonotic disease with 100% lethality and more than 59,000 human deaths annually worldwide^1^. No therapeutic option is available that can reliably revert its fatal consequences accompanied by evident clinical signs^2^.

Current knowledge about rabies pathogenesis and treatments for infected patients indicates that combination therapy using compounds with different action mechanisms has mostly failed in the protocols used to date^2^. Nonetheless, a recent report has shown success using anti-RABV monoclonal antibodies combining intramuscular and intracerebroventricular administration with a micro infusion pump in mice^3^. Another study combined a rabies vaccine with systemic VHH nanobodies, significantly delaying the clinical symptoms in mice and increasing their mean survival time when treated 24 hours after infection^4^.

Camels, alpacas, llamas, and other *Camelidae* species produce both conventional IgG with two heavy chains and two light chains (MW 150 kDa), and IgG2 and IgG3 without light chains, of which the heavy chains have one constant domain (CH1) and one variable domain (VHH), resulting in a molecular weight of ~75 kDa (4 nm × 2.5 nm) called nanobodies (NAbs), with high solubility and specificity, ease of cloning, and thermal and chemical stability^5^.

Due to their small size and low molecular weight, NAbs can access targets that larger antibodies cannot. In therapeutics, nanobodies can be engineered to increase their binding affinity and stability, making them effective in targeting a variety of diseases, including neoplasias and autoimmune disorders. Additionally, their low immunogenicity reduces the risk of adverse immune reactions which is a significant advantage over traditional antibodies^6,7^.

Hence, this study tested the effects of two anti-RABV nanobodies on lethality, incubation period, and viral load in mice previously inoculated with RABV, with the goal of exploring the potential use of these nanobodies as antivirals against rabies.

## MATERIALS AND METHODS

### Ethics

This experiment was approved by the Ethics Committee on Animal Use of the School of Veterinary Medicine and Animal Science (University of Sao Paulo) under protocol Nº 7776070623.

### Virus, anti-RABV conjugate, mice and nanobodies

*In vivo* trials were performed with the RABV CVS (Challenge Virus Standard) strain in BHK-21 cells (10^5^TCID_50%_/mL). A rabbit anti-RABV nucleocapsid IgG conjugated with FITC was kindly provided by Division of Zoonoses Surveillance, Sao Paulo, Sao Paulo State, Brazil.

A total of 40 60-day old male Balb/C mice (Anilab, Paulinia, Brazil) were kept throughout the experiment with feed and water *ad libitum* and a 12 h/12 h light/darkness cycle at the Animal Facility of the Department of Preventive Veterinary Medicine and Animal Health, School of Veterinary Medicine, University of Sao Paulo, Brazil. All mice were anesthetized with isoflurane for all *in vivo* procedures.

INTA (Instituto Nacional de Tecnologia Agropecuaria, Argentina) kindly provided two monoclonal nanobodies (VHH portions only) from llamas (*Lama glama*) against RABV. In short, a nanobody library was produced by vaccinating a llama with the RABV PV strain, antigenically similar to the CVS strain. Total RNA from the vaccinated llama lymphocytes was obtained and cloned into phagemid vectors used to transform *E. coli*. The nanobody library was enriched by three consecutive biopanning rounds through phage display. Finally, the two RABV specific nanobodies were selected by phage ELISA. NAbs identification, total protein concentration and Anti-RABV neutralizing titer were VHH2 = 2.63 mg/mL and 0.87 UI/mL and VHH13 = 2.741 mg/mL and 40.640 UI/mL, respectively.

### Mouse inoculation with RABV and in vivo treatment with nanobodies

All animals were inoculated with 10 µL/nostril of the CVS RABV strain at 10^5^TCID_50%_/mL. Inoculation day was considered as Day (D) 0. At 72 h post-inoculation (D3), mice were treated as follows:

VHH2 Group: 10 mice intracerebrally injected with 40 µL of the combination of 800 µL of VHH2 at 125 µg/mL (in PBS 20 mM Na phosphate, 150 mM NaCl, pH 7.0) in Bioporter^**®**^ (Sigma-Aldrich) as per manufacturer’s instructions.

VHH13 Group: 10 mice intracerebrally injected with 40 µL of the combination of 800 µL of VHH13 at 125 µg/mL (PBS 20 mM Na phosphate, 150 mM NaCl, pH 7.0) in Bioporter (Sigma-Aldrich) as per manufacturer’s instructions.

Control Group: 10 mice intracerebrally injected with 40 µL of PBS 20 mM Na phosphate, 150mM NaCl, pH 7.0.

Bioporter Group: 10 mice intracerebrally injected with 40 µL of the combination of PBS 20 mM Na phosphate 150 mM NaCl, pH 7.0 in Bioporter complex as per manufacturer’s instructions.

Mice were observed daily for signs of the two stages of rabies: stage S-1: ruffled hair and weight loss; stage S-2: ataxia, paralysis, and tremors. Dead mice were frozen at -80 **°**C to preserve the central nervous system (CNS) for subsequent analysis.

### Direct fluorescent antibody test for tissues (dFAT)

Brains of all mice (all groups) were assayed for RABV antigens using the anti-RABV- nucleocapsid FITC-conjugate on acetone-fixed impressions as described^8^.

### Viral load quantification by RT-qPCR

RT-qPCR was carried out with the total RNA extracted from each CNS with PureLink^™^ RNA Mini Kit (ThermoFischer) and the primers JW-12 and N165-146 targeting the N gene^9^. The 20 µL reaction used 1X Power SYBR^®^ Green PCR Master Mix (Applied Biosystems), 125 µM of each primer, 1X RT Enzyme mix, 2 µL of RNA and RNAse-free water in a StepOne^TM^ Real-time PCR System (Applied Biosystems). Amplification conditions were set at 95 °C for 10 min followed by 40 cycles of 94 °C/30 s, 55 °C/30 s and 72 °C/30 s. A melting curve was built after a stage of 95 °C for 15 s and a step of 60 °C for 1 min, with a gradual temperature increase of 0.3 °C up to 95 °C for 15 s.

Viral load quantification in genome copies/µL RNA was performed by RT-qPCR with the absolute quantification method using a standard curve constructed with ten-fold dilution of the pGEM-T Easy plasmid (Promega) with the PV-ptnN (protein N) insert, corresponding to the nucleoprotein of the fixed RABV PV strain as described elsewhere^10^.

Viral load was determined by absolute quantification normalized with the Cq values of the β-actin gene used as endogenous control, with primers described elsewhere^11^. After normalization of the Cq values, the viral load (in number of copies/µL of RNA) of each assay was calculated by the equation of the standard curve line.

### Statistical analysis

Differences in survival times between the four treated groups were tested using the Log-Rank testing Graph R (Version 4.3.2, R Foundation, Vienna, Austria).

## RESULTS

### *In vivo* treatment with nanobodies

#### Survival rates

Mice in groups VHH13, Bioporter, and Control begun to die at D8 and, by D9 (six days after treatment), all were dead; the only difference being that, for the Control group, only two mice died at D8, when compared to three for VHH13 and Bioporter groups.

In the VHH2 group, mice began to die on D9 (n = 9) and the last mouse died on D10, thus representing a 1-day delay in lethality and onset of the first rabies signs compared with the other three groups (Table 1). Mice in this group became ill (stage S-1) only on D8, whereas for the other three groups this occurred on D7. However, we observed no statistically significant difference between the four groups (p = 0.33). All mice that died during the experimental period, both in the treated and control groups, were positive by dFAT.

**Table 1:** RABV genome copy number/µL RNA in the central nervous system (CNS) of mice intranasally inoculated with 10 µL/nostril of the RABV CVS strain at 105.3 TCID50%/mL at D0 and treated with VHH2, VHH13, Bioporter or Control (PBS 20 mM Na phosphate, 150 mM NaCl, pH 7.0.) at D3; *no mice died on this day.

D	VHH2	VHH13	Bioporter	Control
**0**	*	*	*	*
**1**	*	*	*	*
**2**	*	*	*	*
**3**	*	*	*	*
**4**	*	*	*	*
**5**	*	*	*	*
**6**	*	*	*	*
**7**	*	*	*	*
**8**	*	6.9e+08	2.2e+08	3.7e+08
	*	4.4e+08	4.1e+08	5.9e+08
	*	2.6e+08	4.5e+08	*
**9**	2.9e+08	2.0e+08	5.3e+08	5.8e+08
	9.7e+07	2.7e+08	5.5e+08	6.8e+08
	3.7e+08	4.5e+08	4.1e+08	5.5e+08
	1.1e+08	3.4e+08	6.5e+08	4.1e+08
	3.6e+08	3.3e+08	4.6e+08	2.8e+08
	2.9e+08	4.3e+08	4.5e+08	4.8e+08
	8.8e+07	4.6e+08	8.1e+08	3.4e+08
	1.7e+08	*	*	3.3e+08
	8.1e+07	*	*	*
**10**	3.2e+08	*	*	*

#### Viral load quantification by RT-qPCR

**Figure 1:**
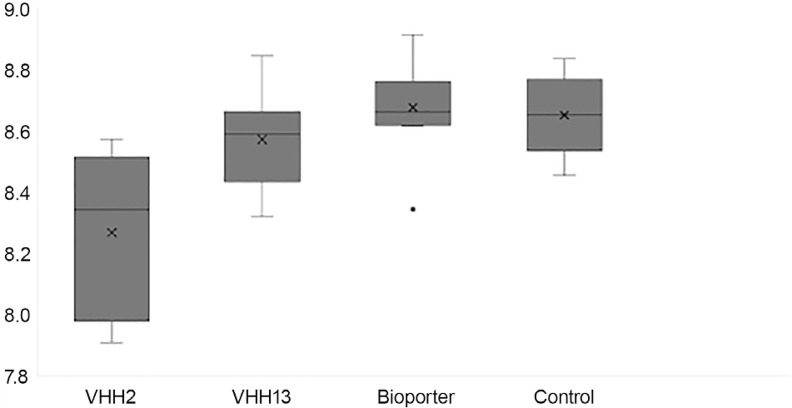
RABV log genome copy number/µL RNA in the central nervous system (CNS) of mice inoculated intranasally with 10 µL/nostril of RABV CVS strain at 105.3 TCID50%/mL on D0 and treated with VHH2, VHH13, Bioporter, or Control (PBS 20 mM sodium phosphate, 150 mM NaCl, pH 7.0) (10 mice/group) at D3.

Mean values of the RABV genome copy numbers/µL RNA for the VHH2-treated group as determined by RT-qPCR was the lowest of all groups at D9 (X– 2.06E+8 sd 1.2E+8) when compared with VHH13 (X– 3.61E+8 sd 9.9E+7), Control (X– 4.6E+8 sd 1.4 E+8) and Bioporter (X– 5.568E+8 sd 1.4 E+8) (Table 1 and Figure 1).

At D8, when mortality started in groups Bioporter, VHH13 and Control, mean and standard deviation of RABV genome copy numbers/µL RNA were, respectively, X– 3.62E+8 sd 1.24 E+8, X– 4.7E+8 sd 2.16 E+8 and X– 4.85E+8 sd 1.61E+8 (Table 1 and Figure 1).

## DISCUSSION

This investigation used two anti-RABV monoclonal nanobodies (NAbs) against RABV to treat mice previously inoculated with the RABV CVS strain; the VHH2 NAbs was able to reduce viral load and delay the onset of signs and lethality.

VHH2 showed a lower RABV neutralizing activity compared with VHH13, but despite this lower anti-G titer, VHH2 may have paratopes against another RABV proteins or even against a non-neutralizing G epitope, which could have accounted for its intracellular impairment of the RABV life cycle.

The CDR3 region of heavy-chain-only antibodies (HcAb) has the unique ability to form long, finger-like extensions that can fit into antigenic cavities, such as the active site of enzymes. These extensions can occupy the substrate cleft in a convex manner, unlike the Fab fragment of conventional monoclonal antibodies which binds to a flat face, away from the active site^12^. Upon binding to an antigen, an antibody can induce allosteric movements in the target protein^13^, thus modifying its shape and possibly affecting its function.

The viral load reduction achieved by VHH2 administration, though not statistically significant, may also be related to its ability to enter cells without the need to form a complex with Bioporter. For instance, VHH-GFP fusion protein, which specifically marked GFAP in mouse brain sections, crossed the blood-brain barrier and labeled astrocytes *in vivo* ^14^.

Studies show that VHH treatment achieved significant protection in mice intranasally infected with CVS when administered 24h post infection, though the virus is still in the olfactory bulb at that moment, and showed no decreased protection five-days post inoculation^5^. Thus, to try to treat mice at a later stage of infection, we chose the 72h post-infection as a mid-point.

A recent study^4^ used a protocol with human monoclonal antibodies administered intramuscularly and intracerebrally that showed 100%, 55.6%, and 33.3% efficiency in controlling rabies infection when started at six, seven, and eight days post inoculation. The efficacy of this approach appears to be related to the simultaneous application of these antibodies intramuscularly and via the intracerebroventricular route, whereas mice treated only with intramuscular cocktails showed no remission in advanced rabies infection^4^. This finding is also supported by failed human treatments using antibodies as part of the protocol for human patients, or in patients who received the protocol with immunoglobulin, vaccine, and antiviral drugs and still died^15^.

As discussed above, anti-rabies VHHs and monoclonal antibodies (mAbs) can intercept the spread of the intracellular virus. However, inhibiting the intracellular virus replication is challenging, as previously shown with the transfection of IgG F(ab)^16^.

A major limitation to this study is that no wild-type RABV strain was used, but only the fixed CVS strain, based on the similarity in antigenicity between this strain and the PV strain used to produce the nanobodies. Whether these nanobodies also react against wild-type RABV strains remains to be determined. Another limitation is that although the mice were treated at a time when RABV was replicating in the CNS, they were not symptomatic and therefore understanding the effect of VHHs at a more advanced rabies stage is still pending.

## CONCLUSION

Nanobody VHH2 reduced RABV virus load but was not sufficient to promote clinical recovery and elimination of rabies virus in the CNS of mice previously inoculated with the CVS RABV strain.

## Data Availability

The complete anonymized dataset supporting the findings of this study is included within the article itself.

## References

[1] World Health Organization Rabies.

[2] Hemachudha T, Ugolini G, Wacharapluesadee S, Sungkarat W, Shuangshoti S, Laothamatas J (2013). Human rabies: neuropathogenesis, diagnosis, and management. Lancet Neurol.

[3] Zeiler F A, Jackson AC (2016). Critical appraisal of the Milwaukee protocol for rabies: this failed approach should be abandoned. Can J Neurol Sci.

[4] Melo GD, Sonthonnax F, Lepousez G, Jouvion G, Minola A, Zatta F (2020). A combination of two human monoclonal antibodies cures symptomatic rabies. EMBO Mol Med.

[5] Terryn S, Francart A, Lamoral S, Hultberg A, Rommelaere H, Wittelsberger A (2014). Protective effect of different anti-rabies virus VHH constructs against rabies disease in mice. PLoS One.

[6] Van Audenhove I, Gettemans J (2016). Nanobodies as versatile tools to understand, diagnose, visualize and treat cancer. EBioMedicine.

[7] Yu T, Zheng F, He W, Muyldermans S, Wen Y (2024). Single domain antibody: development and application in biotechnology and biopharma. Immunol Rev.

[8] Dean DJ, Abelseth MK, Atanasiu P, Meslin FX, Kaplan MM, Koprowski H (1996). Laboratory techniques in rabies.

[9] Hayman DT, Banyard AC, Wakeley PR, Harkess G, Marston D, Wood JL (2011). A universal real-time assay for the detection of Lyssaviruses. J Virol Methods.

[10] Conselheiro JA, Barone GT, Miyagi SA, Silva SO, Agostinho WC, Aguiar J (2022). Evolution of rabies virus isolates: virulence signatures and effects of modulation by neutralizing antibodies. Pathogens.

[11] Ono EA, Taniwaki SA, Brandão P (2017). Short interfering RNAs targeting a vampire-bat related rabies virus phosphoprotein mRNA. Braz J Microbiol.

[12] Wesolowski J, Alzogaray V, Reyelt J, Unger M, Juarez K, Urrutia M (2009). Single domain antibodies: promising experimental and therapeutic tools in infection and immunity. Med Microbiol Immunol.

[13] Al Qaraghuli MM, Kubiak-Ossowska K, Ferro VA, Mulheran PA (2021). Structural analysis of anti-hapten antibodies to identify long-range structural movements induced by hapten binding. Front Moln Biosci.

[14] Li T, Bourgeois JP, Celli S, Glacial F, Le Sourd AM, Mecheri S (2012). Cell-penetrating anti-GFAP VHH and corresponding fluorescent fusion protein VHH-GFP spontaneously cross the blood-brain barrier and specifically recognize astrocytes: Application to brain imaging. FASEB J.

[15] Júnior DS, Marques MS, Oliveira RC (2024). Adapted Milwaukee protocol for rabies treatment in a Brazilian indigenous child: case report. Virol J.

[16] Agostinho WC, Brandão PE (2020). Intracerebral transfection of anti-rabies virus antibodies is an effective therapy for rabies. J Neurovirol.

